# Physiological and Transcriptomic Comparison of Two Sunflower (*Helianthus annuus* L.) Cultivars With High/Low Cadmium Accumulation

**DOI:** 10.3389/fpls.2022.854386

**Published:** 2022-05-09

**Authors:** Yuanzhi Fu, Halyna Zhatova, Yuqing Li, Qiao Liu, Volodymyr Trotsenko, Chengqi Li

**Affiliations:** ^1^School of Life Science and Technology, Henan Institute of Science and Technology, Xinxiang, China; ^2^Faculty of Agrotechnologies and Natural Resource Management, Sumy National Agrarian University, Sumy, Ukraine; ^3^Life Science College, Yuncheng University, Yuncheng, China

**Keywords:** antioxidant, cadmium accumulation, cadmium stress, cadmium tolerance, differential expression genes, transcriptome

## Abstract

The toxic heavy metal cadmium (Cd) is easily absorbed and accumulated in crops and affects human health through the food chains. Sunflower (*Helianthus annuus* L.) is a globally important oil crop. In this study, two sunflower cultivars 62\3 (high Cd) and JB231AC (low Cd), were chosen to compare physiological and transcriptomic responses at different Cd concentrations (0, 25, 50, and 100 μM). The results showed that JB231AC had better Cd tolerance than 62\3. The contents of H_2_O_2_ and MDA (malondialdehyde) in 62\3 were lower than that in JB231AC under Cd stress, but the activities of SOD (superoxide dismutase) and POD (peroxidase) in JB231AC were higher than in 62\3, which indicated that JB231AC had a strong ability to remove reactive oxygen species (ROS)-induced toxic substances. Many deferentially expressed *ABC* (*ATP-binding cassette*) and *ZIP* (*Zn-regulated transporter, Iron-regulated transporter-like protein*) genes indicated that the two gene families might play important roles in different levels of Cd accumulation in the two cultivars. One up-regulated *NRAMP* (*Natural resistance-associated macrophage protein*) gene was identified and had a higher expression level in 62\3. These results provide valuable information to further understand the mechanism of Cd accumulation and provide insights into breeding new low Cd sunflower cultivars.

## Introduction

Cadmium (Cd) is a non-essential heavy metal element in plants and animals, which inhibits plant growth and development, and seriously threatens human health though the food chains (Maria Celeste et al., 2013). With the rapid development of human activities, such as mining, industrial, and agricultural production, heavy metal soil pollution has become increasingly serious because of the release of industrial wastewater, waste gas discharge, sewage irrigation, and misuse of chemical fertilizers and pesticides (Yan et al., 2021). Cd is easily absorbed and accumulated in plants (Shahid et al., 2017; Chen et al., 2018), and soil Cd pollution seriously affects crop quality and safety processes in production. In order to reduce soil Cd pollution in crops, breeders have carried out genetic improvement screening programs to identify soil metal hyperaccumulators and low Cd accumulation cultivars.

Currently, *Sedum alfredii* (Hu et al., 2019), *Solanum nigrum* (Dou et al., 2020), and *Bidens pilosa* (Dou et al., 2019) have been studied extensively as hyperaccumulators based on their ability to grow in Cd-rich soils and to accumulate large amounts of Cd. In addition, cultivating low Cd crop varieties is one of the most effective ways of reducing the human health risk, and a great deal of research has been conducted on crops, such as rice (Liu C. et al., 2020) and wheat (Liu N. et al., 2020). However, there is only limited understanding of the mechanism of Cd detoxification and accumulation in plants, which hinders further development and application of hyperaccumulators and low Cd accumulation cultivars.

At specific concentrations, Cd toxicity can severely affect plant metabolism, respiration, photosynthesis, transport, and growth, which results in reduced root activity, slow seedling development, small and yellow leaves, and eventually leads to plant death (Zhang et al., 2014; Ahmad et al., 2015). Plants respond to Cd stress by adjusting their own physiological and biochemical processes, among which the accumulation and subsequent detoxification of reactive oxygen species (ROS) caused by heavy metals is an important defense response (Zhang F. et al., 2020). In order to reduce the oxidative damage caused by excessive ROS induced by Cd stress, plants have evolved antioxidant enzyme and non-enzyme systems over a long-term phylogenetic process. Key antioxidant enzymes include superoxide dismutase (SOD), peroxidase (POD), catalase (CAT), and glutathione reductase (GR). Non-enzymatic systems mainly consist of reduced glutathione (GSH) and ascorbic acid (AsA) (Wang et al., 2018). Under the regulation of antioxidant enzyme defense systems, plants can maintain normal growth and development within a certain range of heavy metal concentration.

With the advent of next-generation sequencing technologies, many transcriptomic studies on heavy metal stress have focused mainly on rice (Oono et al., 2016), wheat (Zhou et al., 2019), rapeseed (Zhang et al., 2018a), *Sedum alfredii* (Yang et al., 2017), and mustard (Zhang D. et al., 2021), which have revealed the molecular mechanism of transport, accumulation, and detoxification of Cd in different plants. Two rapeseed genotypes were chosen to investigate the Cd translocation mechanism by transcriptomic comparison, and the results showed that *BnNramp2;1* and *BnNramp4;2* were two main Cd transporters (Wang et al., 2019). Hu et al. (2019) compared the transcriptomes of different tissues of *Sedum alfredii* under Cd stress, and the results showed that ATP-binding cassette (ABC) transporters exhibited significant enrichment and accounted for approximately one-third of total selected differentially expressed genes.

Sunflower (*Helianthus annuus* L.) belongs to the *Asteraceae* family and is an important food and bioenergy product (Bashir et al., 2021). It is used in phytoremediation research because of its large biomass, fast growth, and high tolerance to heavy metals (Tang et al., 2003; Bayat et al., 2021; Benavides et al., 2021). However, few Cd accumulation-related studies in physiology and transcriptomics have been conducted with sunflower. In our study, two sunflower cultivars 62\3 and JB231AC were chosen as high/low Cd accumulators and physiological and transcriptomic analyses were conducted under different levels of Cd stress. The study provides important information for further research on Cd accumulation and detoxification mechanisms in sunflower.

## Materials and Methods

### Plant Material and Screening Conditions

Two genotype accessions of sunflower, 62\3 and JB231AC, with high and low Cd accumulation capability were identified in our previous work (data unpublished). The two cultivars were obtained from Sumy National Agrarian University in Ukraine.

In 2020, the healthy seeds of each accession were sterilized by 15% H_2_O_2_ for 30 min, rinsed in distilled water three or more times, and then soaked in deionized water at room temperature for 4 h. Then, they were sown in a germination box (32 cm × 25.5 cm × 11 cm) containing vermiculite and moistened with deionized water. Seeds were incubated in a culture room with 16 h light (28°C, 5,000 Lux) and 8 h dark (25°C) photoperiod for 6 days. Following germination, seedlings were transferred to plastic pots (19 cm × 13 cm × 12 cm) filled with 10 L of 1/4 strength modified Hoagland nutrient solution and grown for 7 days. Then, the nutrient solution was increased to 1/2 strength for 7 days. After 20 days of Cd-free growth, seedlings of uniform size were randomly assigned to four different Cd treatments: 0, 25, 50, and 100 μM CdCl_2_⋅2.5H_2_O for 7 days. The 1/2 strength nutrient solution contained 2.5 mmol/L Ca(NO_3_)_2_, 1 mmol/L MgSO_4_, 0.5 mmol/L NH_4_H_2_PO_4_, 2.5 mmol/L KCL, 2 mmol/L NaCl, 0.2 μmol/L CuSO_4_, 1 μmol/L ZnSO_4_, 0.1 mmol/L EDTA-FeNa, 0.02 mmol/L H_3_BO_3_, 5 nmol/L (NH_4_)_6_Mo_7_O_24_, and, 1 μmol/L MnSO_4_. Finally, the seedlings were harvested; half were used to measure morphological indexes and Cd content, and the remaining half were quickly isolated, frozen in liquid nitrogen, stored at –80°C, and then used to measure physiological indicators and transcriptome analysis. All the treatments were replicated three times.

### Identification of Growth Index and Cadmium Content

For the presentation and evaluation of Cd treatment results, the plants were photographed, and height and fresh weight were measured manually. The roots and shoots of seedlings were oven-dried at 80°C until constant weight, and then weighed for dry weight.

To measure Cd content, the roots of seedlings were rinsed with deionized water at least three times to remove surface ions. Then, roots and shoots were harvested separately. The samples were dried at 105°C for 30 min, and then at 80°C in an oven until completely dry. The dry samples were then ground to a powder. The dry powder for each sample was digested in 5 ml HNO_3_ overnight (at least for 3 h) at room temperature, then 2 ml H_2_O_2_ was added, then further digested for approximately 3 h at 180°C. The digested solution was diluted to 25 mL, and then investigated by an atomic absorption spectrophotometer (ICP-OES, Optima 2100DV, Perkin Elmer, United States).

### Investigation of Antioxidant Systems and the Ascorbate-Glutathione Cycle

To further investigate and compare the physiological characteristics of Cd tolerance of 62\3 and JB231AC, the concentration of hydrogen peroxide (H_2_O_2_) and malondialdehyde (MDA) in the leaves of each fresh sample was measured. Antioxidant enzyme systems were evaluated by detecting the activities of SOD and POD. The Ascorbate-Glutathione (AsA-GSH) cycle was investigated by detecting the activity of glutathione S-transferase (GST) and the concentration of AsA. All physiological indexes were carried out with the corresponding assay kits (Jiancheng Biotechnology, Nanjing, China).

### RNA-Seq Analysis and Library Assembly

Based on the phenotypic results of 62\3 and JB231AC under Cd gradients combined with previous studies, two treatments (0 and 50 μM CdCl_2_⋅2.5H_2_O) of the two cultivars were selected for further transcriptomic analysis. The total RNAs were extracted from leaves for analysis of sequencing libraries by NEBNext^®^Ultra™ RNA Library Prep Kit for Illumina^®^ (NEB, United States), and the library quality was assessed on the Agilent Bioanalyzer 2100 system. The library preparations were sequenced on an Illumina NovaSeq 6000 platform after clustering on a cBot Cluster Generation System using TruSeq PE Cluster Kit v3-cBot-HS (Illumina) in Biomarker Biotechnology Corporation (Beijing, China). To obtain high quality clean data, reads containing adapter, reads containing ploy-N, and low quality reads were removed from raw data. Transcriptome was assembled based on the left.fq and right.fq using Trinity (Grabherr et al., 2011). Fragments per kilobase of transcript per million mapped reads (FPKM) (Trapnell et al., 2010) were used to estimate gene expression level in RNA sequencing.

### Screening of Differential Expression Genes

The DESeq R package (1.10.1) (Love et al., 2014) was used for differential expression analyses of two conditions/groups. Differential expression genes (DEGs) were identified according to the threshold values: FDR < 0.01 and | log_2_ (fold change)| > 2. Gene Ontology (GO) enrichment analysis of the DEGs was implemented by the topGO R packages based on the Kolmogorov–Smirnov test. Kyoto encyclopedia of genes and genomes (KEGG) pathway enrichment was analyzed using KOBAS software to test the statistical enrichment of the DEGs (Mao et al., 2005).

### Validation of Gene Expression by Quantitative Real Time PCR

Twelve RNA samples obtained from RNA-seq experiments were reverse transcribed into cDNA. The qRT-PCR reactions were performed using cDNA as a template in three biological and three technical replicates. The PCR reaction conditions were denaturation at 95°C for 5 min followed by 40 cycles at 95°C for 5s and 60°C for 30s. The specific primers of candidate genes for qRT-PCR were designed by Primer Premier 6.0 software, and the reference gene was 18s rRNA (gene accession No: HM638219) of sunflower (Supplementary Table 1). Relative expression was analyzed using the cycle threshold 2^–ΔΔCt^ method (Livak and Schmittgen, 2001).

### Statistical Analysis

Transfer factor (TF) is the ratio of the concentration of Cd in the shoot to that in the root. All data were statistically analyzed using GraphPad Prism 8. Two-way ANOVA was performed on data sets, and the mean and standard deviation (SD) of each treatment was calculated. Multiple comparisons with Sidak’s test were mainly used to compare the mean values between treatments (*p* < 0.05).

## Results

### Comparison of Phenotype Characteristics to Cadmium Stress Between High/Low Cadmium Sunflower Cultivars

**FIGURE 1:**
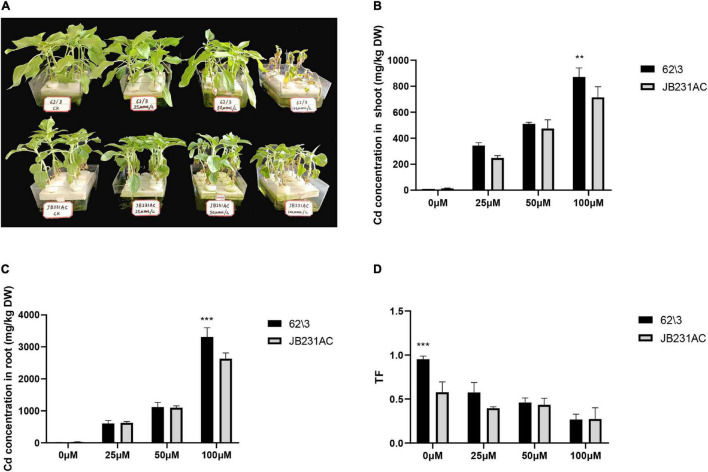
Performance of growth and Cd accumulation of 62\3 and JB231AC genotypes. **(A)** Growth performance of 62\3 and JB231AC grown hydroponically under Cd stress for 7 days. **(B)** Cd concentration in the shoots of the seedlings. **(C)** Cd concentration in the roots of the seedlings. **(D)** TF (Translocation factor), the ratio of Cd concentration in shoots to that in roots. For **(B–D)** the statistical analyses were conducted using all the performance data of 62\3 and JB231AC under Cd stress. Data presented are the means (*n* = 3), and error bars denote the standard deviations. The asterisk represent the significant difference between 62\3 and JB231AC under one same Cd stress. **P < 0.01, ***P < 0.001.

Plant growth parameters were investigated in order to examine the plant growth differences between 62\3 and JB231AC under Cd stress. With an increase in Cd concentration, the seedlings of both cultivars showed gradually weaker growth potential and became yellow (Figure 1A), and 62\3 almost wilted and died under 100 μM Cd stress. The result showed that 62\3 was sensitive to Cd toxicity; i.e., JB231AC had a higher Cd tolerance than 62\ 3.

The Cd concentration in the roots was much higher than in the shoots of both cultivars, and the Cd concentration in the roots and shoots of 62\3 was higher than in JB231AC, particularly under 100 μM Cd stress (Figures 1B,C). The Cd TF value of 62\3 was higher than JB231AC in the 0–50 μM range of Cd concentration, and there was no significant difference between the two cultivars under the same concentration of Cd stress (Figure 1D). The result showed that 62\3 was a high Cd accumulation cultivar and had a higher root-to-shoot Cd translocation ratio than JB231AC.

**FIGURE 2:**
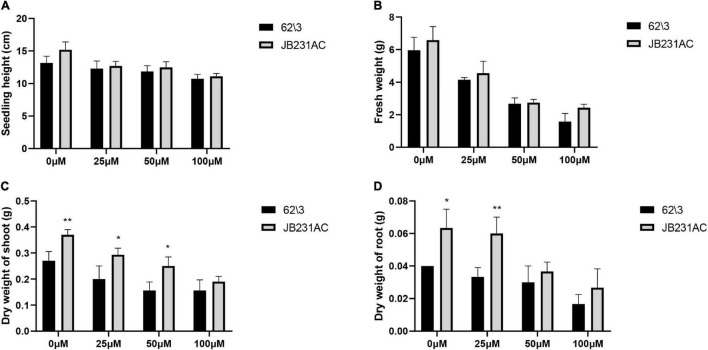
Vegetative performance of 62\3 and JB231AC. **(A–D)** Growth-related indices of the seedlings, seedling height **(A)**, fresh weigh **(B)**, dry weight of shoot **(C)** and dry weight of root **(D)**. For **(A–D)** the statistical analyses were conducted using all the performance data of 62\3 and JB231AC under Cd stress. Data presented are the means (*n* = 3), and error bars denote the standard deviations. The asterisk represent the significant difference between 62\3 and JB231AC under one same Cd stress. *P < 0.05, **P < 0.01.

The seedling height and fresh weight of 62\3 and JB231AC decreased with an increase in Cd concentration, but there was no difference between the two cultivars at the same concentration of Cd stress (Figures 2A,B). The dry weight of shoots and roots of 62\3 and JB231AC decreased with an increase in Cd stress, but the genotype JB231AC had a larger biomass than 62\3 at the same concentration of Cd stress (Figures 2C,D).

### Physiological Responses to Cadmium Stress in 62\3 and JB231AC

**FIGURE 3:**
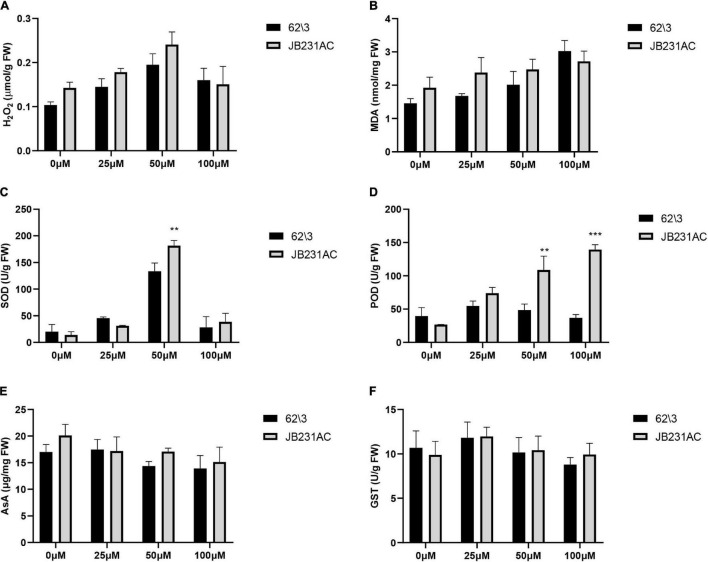
Antioxidant systems of 62\3 and JB231AC under Cd stress. **(A)** H_2_O_2_ content. **(B)** MDA, Malondialdehyde. **(C)** SOD, Superoxide dismutase. **(D)** POD, Peroxidase. **(E)** AsA, Ascorbic acid. **(F)** GST, Glutathione S-transferase. For **(A–F)** the statistical analyses were conducted using all the performance data of 62\3 and JB231AC under Cd stress. Data presented are the means (*n* = 3), and error bars denote the standard deviations. The asterisk represent the significant difference between 62\3 and JB231AC under one same Cd stress. **: *P* < 0.01, ***: *P* < 0.001.

The contents of H_2_O_2_ and MDA were measured to investigate the level of antioxidant reaction to Cd stress. The H_2_O_2_ content was higher in JB231AC than in 62\3 in the 0–50 μM range of Cd stress; it increased with an increase in Cd concentration, and then decreased under 100 μM Cd stress (Figure 3A). The content of MDA increased with an increase in Cd concentration and was higher in JB231AC than in 62\3 in the 0–50 μM range of Cd stress (Figure 3B). The contents of H_2_O_2_ and MDA were not significantly different between 62\3 and JB231AC under the same concentration of Cd stress.

The activities of SOD and POD were important indices for showing Cd tolerance levels in antioxidant enzyme systems. With an increase in Cd concentration, the activity of SOD first increased quickly and then decreased quickly, and reached a maximum value under 50 μM Cd stress with significant differences seen between 62\3 and JB231AC (Figure 3C). This result indicated that the activity of SOD was strongly stimulated under 50 μM Cd stress. With an increase in Cd concentration, the activity of POD in 62\3 first increased and then decreased, and reached a maximum value under 25 μM Cd stress (Figure 3D). However, the activity of POD in JB231AC increased with an increased Cd concentration. The activity of POD was higher in JB231AC than in 62\3, and showed highly significant differences under 50 μM and 100 μM Cd stress. This result indicated that the regulation response of POD to Cd stress was different in the two cultivars.

The changes in concentration of AsA and the activities of GST were almost unaffected at different Cd concentrations compared to the control, and there was no significant difference between cultivars (Figures 3E,F). This result suggested that there was negligible AsA and GST response to Cd stress in this study.

### RNA-Seq Analyses of 62\3 and JB231AC Under Cadmium Stress

The leaves of the no-Cd control and Cd-treated (50 μM CdCl_2_⋅2.5H_2_O) plants were collected to investigate molecular responses at the transcriptional level by RNA-seq. Twelve samples (containing three replicates per treatment) were processed for mRNA sequencing, and 93.62 Gb clean data with Q30 ≥ 93.90% was obtained (Supplementary Table 2). The data was deposited in the NCBI database (The accession number of Bioproject is PRJNA797513 and the accession number of Biosample is SAMN25008009). A total of 78,975 unigenes with a total length of 59,469,664 nt and an average length of 1,340 nt were obtained after sequence assembly (Supplementary Table 3). Length distribution of unigenes and saturation test of RNA-seq data were all perfect (Supplementary Figures 1, 2). The result of sequencing and assembly showed that the mRNA-seq Library was of high quality and could be used for transcriptome data analyses.

### Identification of Cadmium-Regulated Differential Expression Genes

**FIGURE 4:**
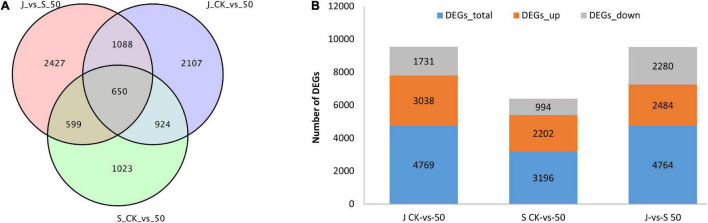
DEGs comparison of 62\3 and JB231AC. **(A)** Venn diagrams showing all DEGs shared among the three groups. **(B)** Number of DEGs that were up or down-regulated in the three groups. S: 62\3, J: JB231AC, CK: 0 μM CdCl_2_⋅2.5H_2_O, 50: 50 μM CdCl_2_⋅2.5H_2_O, vs., versus.

To further elucidate the molecular mechanisms involved in Cd tolerance, the high-throughput DEGs sequencing between 62\3 and JB231AC under Cd stress was performed by DESeq R package (1.10.1). Fold change (FC) ≥ 2 and the criteria of false discovery rate (FDR) < 0.01 were the threshold values. A total of 8,818 DEGs were identified, and among them, 3,196, 4,769, and 4,764 DEGs were identified in the three comparison groups 62\3 (CK vs. 50 μM), JB231AC (CK vs. 50 μM), and 62\3 vs. JB231AC (50 μM Cd), respectively (Figure 4). There were 650 co-expressed DEGs in the three groups. More DEGs were identified in JB231AC (CK vs. 50 μM) than in 62\3 (CK vs. 50 μM), and the two cultivars had more up-regulated DEGs than down-regulated. Comparing 62\3 with JB231AC under 50 μM Cd stress, there were approximately equal numbers of up- and down-regulated DEGs.

### Functional Annotations of Cadmium-Regulated Differential Expression Genes

**FIGURE 5:**
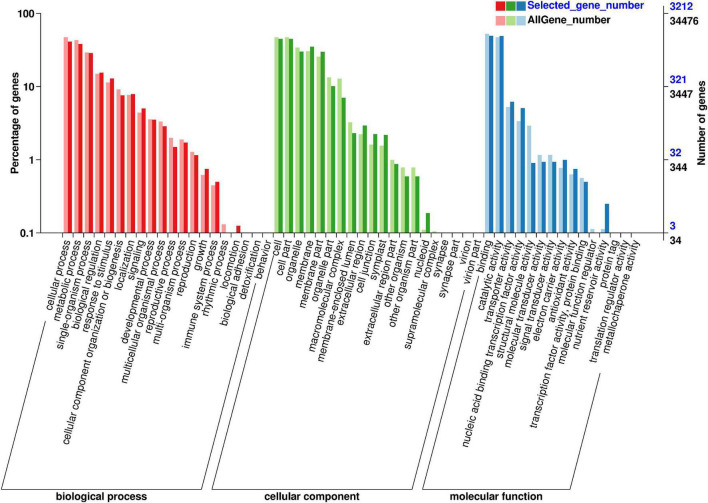
GO classifications of DEGs of 62\3 and JB231AC under 50 μM Cd stress.

GO class analysis was used to identify the function annotation of 4,764 DEGs between 62\3 and JB231AC under 50 μM Cd stress (Figure 5 and Supplementary Table 4). The result showed that 55 GO terms were grouped into three categories (biological process, cellular component, and molecular function). Binding and catalytic activity had the highest DEG number, followed by cell, cell part, cellular process, and metabolic process.

**FIGURE 6:**
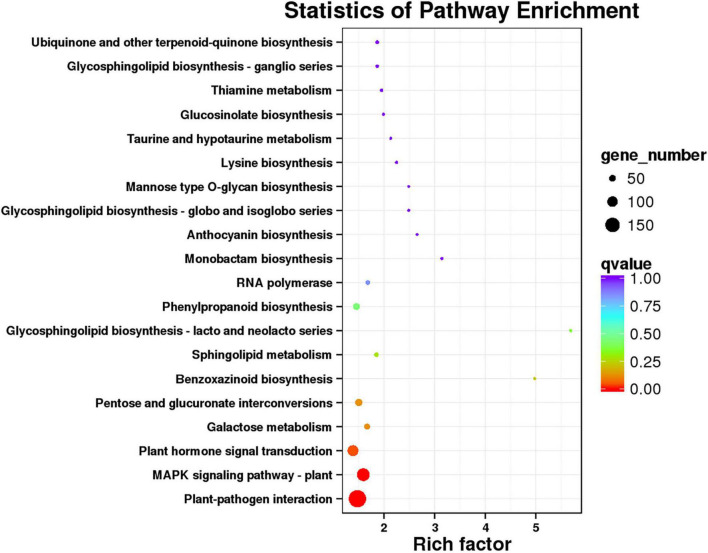
KEGG pathways of DEGs of 62\3 and JB231AC under 50 μM Cd stress.

In order to identify the metabolic pathway enrichment between 62\3 and JB231AC under 50 μM Cd stress, 4,764 DEGs were analyzed by KEGG. A total of 130 KEGG pathways were enriched and 20 KEGG pathways were significantly enriched (Figure 6 and Supplementary Table 5). Among them, plant-pathogen interaction, MAPK signaling pathway-plant, plant hormone signal transduction, galactose metabolism, and pentose and glucuronate interconversions were listed in the top five KEGG pathways.

### Differential Expression Genes Related to Cadmium Detoxification, Transport, and Accumulation

**FIGURE 7:**
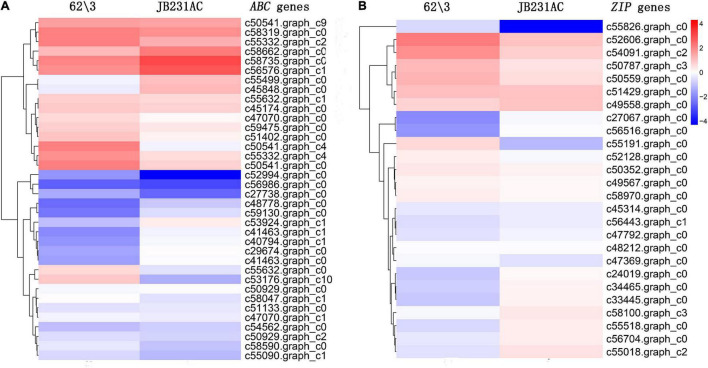
Cluster heat maps of DEG expression of 62\3 and JB231AC under 50 μM Cd stress. **(A)** Predicted *ATP-binding cassette* (*ABC*) gene expression. **(B)** Predicted *ZIP (Zn-regulated transporter, Iron-regulated transporter-like protein)* gene expression. Expression values of two cultivars were presented as log_2_-transformed normalized FPKM (Fragments Per Kilobase of transcript per Million mapped reads) values, and FPKM value was average of three replicates. Additional details are presented in Supplementary Tables 6, 7.

A total of 79 DEGs related to Cd detoxification, transport, or accumulation were identified. Among them, 36 DEGs *ABC*, 24 up-regulated, and 12 down-regulated, were identified between 62\3 and JB231AC under 50 μM Cd stress, accounting for 43.4% of all selected Cd related genes (Figure 7A and Supplementary Table 6). Both DEGs, *c50541. graph_c4* (log_2_FC = 4.987981385) and *c53176. graph_c10* (log_2_FC = 4.848310244), were up-regulated and had a higher differential expression in 62\3 than in JB231AC.

Twenty-six DEGs *Zn-regulated transporter, Iron-regulated transporter-like protein* (*ZIP*), 15 up-regulated, and 11 down-regulated, were identified in 62\3 and JB231AC under 50 μM Cd stress, accounting for 31.3% of all selected Cd related genes (Figure 7B and Supplementary Table 7). Among them, the genes *c55191.graph_c0* (log_2_FC = 6.693782781) and *c55826.graph_c0* (log_2_FC = 4.498795) were up-regulated and had higher differential expressions in 62\3 than in JB231AC.

In addition, Cd-related DEGs, including *HIPP* (*Heavy metal associated isoprenylated plant protein*), *MTP* (*Metal tolerance protein*), *YSL* (*Metal-nicotianamine transporter*), *HMA* (*Heavy metal ATPase*), and *NRAMP* (*Natural resistance-associated macrophage protein*), were screened out, and the number of DEGs for each was 7, 4, 3, 2, and 1, respectively (Supplementary Table 8). Among them, there were 12 up-regulated and 5 down-regulated, with a total of 17. The up-regulated gene *c37848.graph_c0*, belonging to the *HIPP* family, had a large differential expression level (Log_2_FC = 4.28146756), and its expression level in 62\3 was higher than in JB231AC. One up-regulated *NRAMP* gene, *c53780.graph_c0* (Log_2_FC = 4.207403041), was identified and had a higher expression level in 62\3. Two up-regulated *HMA* genes, *c55845.graph_c0* and *c49575.graph_c0*, were screened out and both had a higher expression levels in 62\3 than in JB231AC.

### Validation of Differential Expression Genes by Quantitative Real Time PCR

**FIGURE 8:**
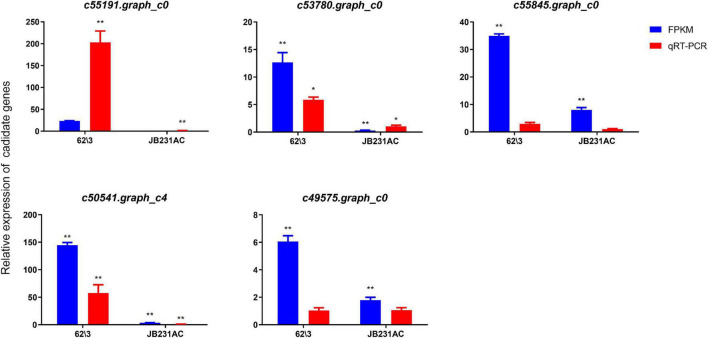
RNA-Seq and qRT-PCR were used to compare DEGs in 62\3 and JB231AC. The relative gene expression level was calculated by 2^–ΔΔCT^ in qRT-PCR. *P < 0.05, **P < 0.01.

To verify the expression levels of DEGs identified by RNA-seq, five Cd-induced key genes used for Quantitative Real Time PCR (qRT-PCR) verification were randomly selected from the comparison group of the two cultivars with Cd treatment (50 μM). As a whole, the results showed that the expression profile of qRT-PCR of 5 candidate genes were consistent with the trend of their transcriptome data, most up-regulated expression (Figure 8). Therefore, The RNA-seq data and transcriptome results are mostly reliable and contribute to the identification of DEGs related to Cd.

## Discussion

### Growth and Physiological Response of Two Sunflower Cultivars to Cadmium Stress

In this study, the two sunflower cultivars 62\3 and JB231AC were selected as high and low Cd accumulation cultivars for physiological study under Cd stress. The Cd concentration in both roots and shoots of 62\3 was higher than that in JB231AC, and root-to-shoot Cd translocation was stronger in 62\3 than in JB231AC (Figures 1B–D). With increasing Cd stress, JB231AC cultivars became weak and stunted and the leaves turned slightly yellow (Figure 1A). Cultivar 62\3 was sensitive to Cd toxicity and almost withered and died under 100 μM Cd stress. The results show that JB231AC had some tolerance to Cd toxicity. Chen et al. (2021) showed that after 10 days of Cd treatment, plant height and biomass of kenaf seedlings were significantly inhibited under 10 mg L^–1^ Cd stress compared to the control. Saidi et al. (2021) also showed that plant growth was significantly impacted by an increasing Cd dose, and was severely suppressed at the highest Cd concentrations (50 and 100 μM) after Cd treatment for 4 days. Therefore, a 50 μM Cd dose can be considered as an important candidate or reference concentration for studying short-term Cd treatment at the seedling stage.

Cd is an environmental pollutant which increases ROS production, and this leads to excessive accumulation of O_2_, H_2_O_2_, and hydroxyl radicals. This, in turn, leads to oxidation bursts (Heyno et al., 2008) and eventually causes high toxicity in plants (Li et al., 2012; Unsal et al., 2020). Plants avoid or reduce heavy metal-induced oxidative damage by a series of complex enzymatic and non-enzymatic antioxidant defense mechanisms (Yu et al., 2013; Liu J. et al., 2019); i.e., Cd induces membrane lipid peroxidation and regulates the expression of antioxidant defense systems (Chen et al., 2010; Imran et al., 2020). Li et al. (2012) showed that H_2_O_2_ and MDA contents are important indexes to evaluate the degree of peroxidation damage under Cd stress due to ROS-induced membrane lipid peroxidation. In our study, the contents of H_2_O_2_ and MDA in 62\3 was lower than in JB231AC, which showed that JB231AC produces more toxic peroxidation substances (Figures 3A,B). The contents of H_2_O_2_ and MDA in both cultivars increased with an increase in Cd concentration, indicating that oxidative damage was increasingly severe; our findings are similar to the results of other studies (Shi et al., 2013; Kanu et al., 2019; Khan et al., 2020).

SOD and POD are two important antioxidant enzymes that remove ROS (Wang et al., 2019). In our study, the activities of SOD and POD in JB231AC were higher than in 62\3, indicating that JB231AC had a pronounced ability to remove ROS-induced toxic substances. JB231AC had a better tolerance to Cd toxicity and had a significantly higher dry weight of roots and stems than 62\3 (Figures 2C,D). With an increase in Cd concentration, the SOD activity first increased rapidly and then decreased (Figure 3C), which might indicate that SOD in the two sunflower cultivars had a strong response to Cd stress and played an important role in removing harmful products. The variation trend of POD activity was different (Figure 3D), and the regulation mechanism of POD should be different in the two cultivars. The result showed that antioxidant enzymes played important roles in Cd accumulation and tolerance. However, Zhang et al. (2015) found that the antioxidant enzymes governing Cd detoxification were not found to be active in castor leaves under two different Cd concentrations (2 mg L^–1^ and 5 mg L^–1^) over 10 days. The roles of antioxidant enzymes and the AsA-GSH cycle in different materials are very different under Cd stress (Wu et al., 2015). In our study, no differences were found in the AsA content or the activity of GST between the two cultivars under the same Cd treatments (Figures 3E,F), suggesting that the AsA-GSH cycle was not the key factor affecting the differences in Cd accumulation and tolerance between the two cultivars.

### Differential Expression Gene Annotation Functions of Two Sunflower Genotypes Under Cadmium Stress

Many genes are regulated and expressed under Cd stress, and the regulations of Cd-related genes in different Cd accumulation materials are different, which may be the reason for the different Cd accumulations observed. A total of 3,321 and 2,221 DEGs were regulated in the two tolerant and sensitive Cd-treated kenaf cultivars compared to the controls (Chen et al., 2021). The transcriptomic analysis revealed 883 DEGs between control and Cd-stressed plants in *Nicotiana rustica* (high Cd), while 2,119 DEGs were found in *Nicotiana tabacum* (low Cd) (Zhang Y. et al., 2021). In our study, 4,764 genes were differentially expressed by DEGseq between 62\3 and JB231AC under 50 μM Cd stress (Figure 4). GO class showed that most DEGs were classed in binding, catalytic activity, and cell at second level (Figure 5 and Supplementary Table 4), and KEGG enrichment pathways were mostly plant-pathogen interaction, MAPK (mitogen-activated protein kinase) signaling pathway-plant, and plant hormone signal transduction (Figure 6 and Supplementary Table 5). The results indicate that the differences in Cd accumulation between the two cultivars may be caused mainly by DEGs regulation in the above annotations or pathways.

### Key Candidate Genes May Be Involved in Cadmium Detoxification, Transport, and Accumulation

The ABC transporter is located on the vacuole membrane, and can sequester Cd and its complexes in the vacuole (Mendoza-Cózatl et al., 2011). ABC transporters involved in Cd or Cd conjugated transport have been found in some plants, such as *Arabidopsis* (Kim et al., 2007; Park et al., 2012), rice (Oda et al., 2011; Cai et al., 2021), and rapeseed (Zhang et al., 2018b). Spatial expression analysis showed that *OsABCG36* was expressed in the root tip and the mature root region, and knockout of *OsABCG36* increased Cd accumulation in root cell sap and enhanced Cd sensitivity (Fu et al., 2019). Overexpressing *OsABCG48* in rice lowers root Cd accumulation which prevents further transport of Cd to the shoots and seeds (Cai et al., 2021). In our study, of 36 *ABC* DEGs, two out of three genes up-regulated, and were identified in 62\3 and JB231AC under 50 μM Cd stress, accounting for 43.4% of all selected Cd related genes (Figure 7A and Supplementary Table 6). The result showed that ABC transporters probably play a significant role in the regulation of different Cd accumulation in both cultivars.

The plant ZIP family proteins, which belong to integral membrane transporters, exist widely in plants and have the function of transporting various metal elements, such as Zn^2+^, Ca^2+^, Fe^2+^, and Cd^2+^ (Guerinot, 2000; Mills et al., 2005). OsZIP1 is a metal-detoxified transporter preventing excess Cd accumulation in rice. It is located in the endoplasmic reticulum and plasma membrane, it is abundantly expressed in roots, and it is a metal-detoxified transporter by preventing excess Zn, Cu, and Cd accumulation in rice (Liu X. S. et al., 2019). Zheng et al. (2018) showed that there might be a feedback regulation of *OsZIP1* in roots to prevent increasing Cd uptake from soil. Overexpressing *OsZIP1* improves rice growth under excess metal stress but accumulates less of the metals (Liu X. S. et al., 2019). The expression of *OsZIP1* and *OsZIP3* in yeast leads to increased Cd sensitivity and Cd accumulation (Zheng et al., 2018). *OsZIP7*, expressed in parenchyma cells of vascular bundles in roots and nodes, plays an important role in root xylem load and inter-node vascular transport, and its expression promotes Cd transport to shoots (Tan et al., 2019). In our study, of 26 *ZIP* DEGs, 57.7% genes up-regulated, and were identified in 62\3 and JB231AC under 50 μM Cd stress, accounting for 31.3% of all selected Cd related genes (Figure 7B and Supplementary Table 7). Therefore, the ZIP family could play an important role in our findings.

In previous studies, *HMA* (Takahashi et al., 2012; Liu C. L. et al., 2020) and *NRAMP* (Takahashi et al., 2011; Yang et al., 2019) have been shown to be two important Cd transport and detoxification related genes in crops. *OsHMA2* is localized in the pericycle of roots and the phloem of diffuse vascular bundles of nodules, and it is the main transporter of Zn and Cd from the roots to the shoots (Yamaji et al., 2013). *OsHMA3*, expressed in root cell vacuoles, confers high root-to-shoot Cd translocation rates (Miyadate et al., 2011) and it restricts Cd translocation from roots to above-ground tissues by selectively isolating Cd into root vacuoles (Ueno et al., 2010; Miyadate et al., 2011). *NRAMP* family genes are responsible for the transport of divalent cations (Fe^2+^, Zn^2+^, Mn^2+^, and Cd^2+^) (Nevo and Nelson, 2006). *SaNRAMP1* is localized at the plasma membrane, and overexpressing of *SaNRAMP1* in tobacco (*Nicotiana* sp.) significantly increases Cd, Zn, and Mn concentration in the shoots (Zhang J. et al., 2020). In our study, two up-regulated *HMA* genes and one up-regulated *NRAMP* gene were screened out, and they all had a higher expression level in 62\3 than in JB231AC. It is speculated that *HMA* and *NRAMP* might make certain contributes in the transport and accumulation of Cd in the two sunflower cultivars (Supplementary Table 8).

HIPP plays crucial roles in metal homeostasis and detoxification as metal-binding metallochaperones (Khan et al., 2019). *HIPP1-V* was up-regulated in *H. villosa* under Cd stress, and overexpressing *HIPP1-V* showed enhanced Cd tolerance in wheat (Zhang H. et al., 2020). MTPs are divalent cation transporters which are essential for metal homeostasis and tolerance in plants, sequestrating Zn and Cd in vacuoles in cucumber (Migocka et al., 2015). SnYSL3 is a transporter delivering metal-nicotianamine complexes, and *SnYSL3* transgenic plants increase the translocation ratios of Fe and Cd from roots to shoots (Feng et al., 2017). In our study, DEGs related to Cd transport or detoxification, including *HIPP*, *MTP*, and *YSL* were screened out, with 7, 4, and 3, respectively (Supplementary Table 8). This result suggests that HIPP and MTP might perform Cd detoxification and tolerance in the two sunflower cultivars, and YSL might be involved in Cd translocation.

In conclusion, JB231AC has the characteristics of good Cd tolerance and low Cd accumulation, and it can be used as a good germplasm material for hybridization in order to improve the Cd tolerance of sunflower. In follow-up work, the functions of candidate genes related to Cd physiology would be further validated by the genetic transformation, and the functional molecular markers could be developed for the marker-assisted selection of sunflower in future breeding programs.

## Conclusion

Two sunflower cultivars, 62\3 and JB231AC, were verified as high and low Cd accumulators, respectively, and 62\3 had the stronger root-to-shoot Cd translocation ability. Comparative physiological and transcriptomic analyses between the two cultivars revealed that JB231AC had better Cd tolerance. The contents of H_2_O_2_ and MDA in 62\3 were lower than that in JB231AC under Cd stress, which showed that JB231AC produces more toxic peroxidation substances. However, the activities of SOD and POD in JB231AC were higher than in 62\3, indicating that JB231AC had a very strong ability to remove ROS-induced toxic substances. The activity of SOD was simultaneously induced quickly with an increase in Cd stress in the two cultivars, but the variation trend of POD activity was different. The transcriptomic analysis showed that many *ABC* and *ZIP* genes were differentially expressed, indicating that the two kinds of genes might play an important role in different Cd accumulation in the two cultivars. Two up-regulated *HMA* (*Heavy metal ATPase*) genes and one up-regulated *NRAMP* (*Natural resistance-associated macrophage protein*) genes were screened out.

## Data Availability Statement

The data was deposited in the NCBI database (The accession number of Bioproject is PRJNA797513 and the accession number of Biosample is SAMN25008009).

## Author Contributions

YF wrote the manuscript. HZ, YL, and QL performed the experiments. CL and VT designed the experiment. All authors commented on the manuscript.

## Conflict of Interest

The authors declare that the research was conducted in the absence of any commercial or financial relationships that could be construed as a potential conflict of interest.

## Publisher’s Note

All claims expressed in this article are solely those of the authors and do not necessarily represent those of their affiliated organizations, or those of the publisher, the editors and the reviewers. Any product that may be evaluated in this article, or claim that may be made by its manufacturer, is not guaranteed or endorsed by the publisher.

## Supplementary Material

Supplementary Figure 1 Length distribution of unigenes.

Supplementary Figure 2 Express gene saturation test of RNA-Seq data.

Supplementary Table 1 Primers used for qRT-PCR.

Supplementary Table 2 Quality assessment of the raw data.

Supplementary Table 3 Quality of assembled unigenes.

Supplementary Table 4 All GO classifications of DEGs between 62\3 and JB231AC under 50μM Cd stress.

Supplementary Table 5 All KGEE enrichment pathways of DEGs between 62\3 and JB231AC under 50μM Cd stress.

Supplementary Table 6 Details of DEGs predicted to belong to the ATP-binding cassette (ABC) transporter family.

Supplementary Table 7 Details of DEGs predicted to belong to the Zn-regulated transporter, iron-regulated transporter-like protein (ZIP) family.

Supplementary Table 8 Details of differentially-expressed genes (DEGs) predicted to the other Cd-related genes.
