# Supplementary figures and images for: Physiological and Transcriptomic Comparison of Two Sunflower (Helianthus annuus L.) Cultivars With High/Low Cadmium Accumulation
Source: Front Plant Sci. 2022 May 9;13:854386. doi: 10.3389/fpls.2022.854386 (PMC9125308; doi:10.3389/fpls.2022.854386)

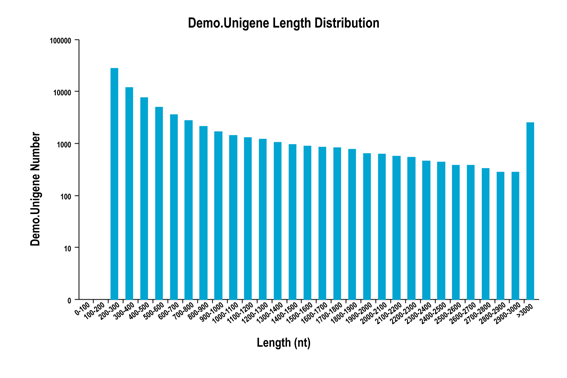

Supplement: Supplementary Figure 1 — Length distribution of unigenes. [file Image_1.TIF]

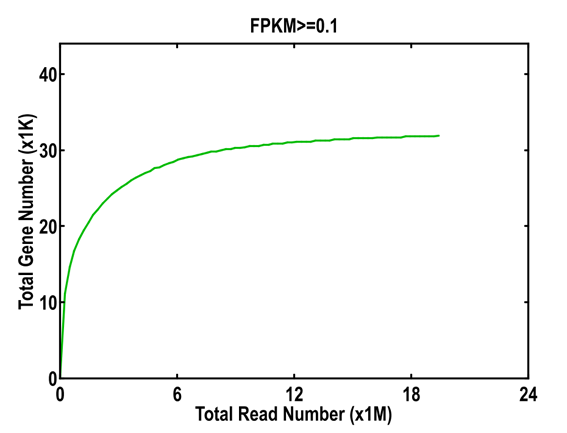

Supplement: Supplementary Figure 2 — Express gene saturation test of RNA-Seq data. [file Image_2.TIF]
